# Lamivudine 24-month-long prophylaxis is a safe and efficient choice for the prevention of hepatitis B virus reactivation in HBsAg-negative/HBcAb-positive patients with advanced DLBCL undergoing upfront R-CHOP-21

**DOI:** 10.3389/fonc.2023.1130899

**Published:** 2023-02-15

**Authors:** Claudia Giordano, Marco Picardi, Novella Pugliese, Annamaria Vincenzi, Davide Pio Abagnale, Laura De Fazio, Maria Luisa Giannattasio, Carmina Fatigati, Mauro Ciriello, Alessia Salemme, Giada Muccioli Casadei, Elena Vigliar, Massimo Mascolo, Giancarlo Troncone, Fabrizio Pane

**Affiliations:** ^1^ Department of Clinical Medicine and Surgery, Federico II University Medical School, Naples, Italy; ^2^ Department of Public Health, Federico II University Medical School Naples, Naples, Italy; ^3^ Department of Advanced Biomedical Sciences, Federico II University Medical School, Naples, Italy

**Keywords:** occult hepatitis B virus infection, non-hodgkin lymphoma, R-CHOP-21, chemotherapy disruption, antiviral prophylaxis

## Abstract

**Introduction:**

Occult hepatitis B infection (OBI) is a condition where replication-competent hepatitis B virus-DNA (HBV-DNA) is present in the liver, with or without HBV-DNA in the blood [<200 international units (IU)/ml or absent] in HB surface antigen (HBsAg)-negative/HB core antibody (HBcAb)-positive individuals. In patients with advanced stage diffuse large B-cell lymphoma (DLBCL) undergoing 6 cycles of R-CHOP-21+2 additional R, OBI reactivation is a frequent and severe complication. There is no consensus among recent guidelines on whether a pre-emptive approach or primary antiviral prophylaxis is the best solution in this setting of patients. In addition, questions still unresolved are the type of prophylactic drug against HBV and adequate prophylaxis duration.

**Methods:**

In this case-cohort study, we compared a prospective series of 31 HBsAg−/HBcAb+ patients with newly diagnosed high-risk DLBCL receiving lamivudine (LAM) prophylaxis 1 week before R-CHOP-21+2R until 18 months after (24-month LAM series) versus 96 HBsAg−/HBcAb+ patients (from January 2005 to December 2011) undergoing a pre-emptive approach (pre-emptive cohort) and versus 60 HBsAg−/HBcAb+ patients, from January 2012 to December 2017, receiving LAM prophylaxis [1 week before immunochemotherapy (ICHT) start until 6 months after] (12-month LAM cohort). Efficacy analysis focused primarily on ICHT disruption and secondarily on OBI reactivation and/or acute hepatitis.

**Results:**

In the 24-month LAM series and in the 12-month LAM cohort, there were no episodes of ICHT disruption versus 7% in the pre-emptive cohort (*P* = 0.05). OBI reactivation did not occur in any of the 31 patients in the 24-month LAM series versus 7 out of 60 patients (10%) in the 12-month LAM cohort or 12 out of 96 (12%) patients in the pre-emptive cohort (*P* = 0.04, by *χ* ^2^ test). No patients in the 24-month LAM series developed acute hepatitis compared with three in the 12-month LAM cohort and six in the pre-emptive cohort.

**Discussion:**

This is the first study collecting data regarding a consistent and homogeneous large sample of 187 HBsAg−/HBcAb+ patients undergoing standard R-CHOP-21 for aggressive lymphoma. In our study, 24-month-long prophylaxis with LAM appears to be the most effective approach with a null risk of OBI reactivation, hepatitis flare-up, and ICHT disruption.

## Introduction

Occult hepatitis B infection (OBI) refers to a condition where replication-competent hepatitis B virus (HBV)-DNA is present in the liver, with or without HBV-DNA in the blood [<200 international units (IU)/ml or absent] in HB surface antigen-negative/HB core antibody-positive (HBsAg−/HBcAb+) individuals (1). The characteristics are the absence of HBV-DNA (or eventually the transient presence of very low levels of viremia) in the serum and the persistence in the liver of the “covalently closed circular DNA” (cccDNA), a long-lasting HBV replication intermediate that can be revealed only by very sensitive techniques like “nested-PCR,” performed on liver tissue (1, 2).

The cccDNA as a chromatinized viral mini chromosome is very stable and long-lasting in the nucleus of infected hepatocytes, and together with the long half-life of hepatocytes, these characteristics facilitate HBV infection that can continue for life (3, 4).

The gold standard for the diagnosis of OBI is the detection of HBV-DNA in the liver of HBsAg-negative individuals; however, standardized and valid assays for HBV-DNA detection in the liver are not available yet (1). Studies using in-house assays have variable sensitivities and specificities. The recommended methods include nested PCR techniques to amplify at least three different viral genomic regions, real-time PCR assays, or droplet digital PCR assays (2, 5). For these reasons, detection in the blood of HBV-DNA is commonly used. HBV-DNA assays can vary and assays with inadequate sensitivity can result in false-negative HBV-DNA results and may lead to a missed diagnosis of OBI. The lower limit of detection of most currently available commercial HBV-DNA assays is 10–20 IU/ml.

HBV is an “elusive” infection, whose real prevalence in the general population is not known, being quite variable depending on a number of factors that can influence the rates of OBI including sampling issues, assay sensitivity, and the prevalence of HBsAg in the geographical region in which the study was conducted (6, 7).

However, several studies report that the prevalence of OBI is approximately 16%–18% in subjects with evidence of previous HBV infection (i.e., HBsAg−/HBcAb+ patients) and 7%–8% in subjects totally seronegative for HBV (8, 9).

OBI reactivation can occur in up to 40% of patients treated with potent immunosuppressive regimens (10–12). In particular, in diffuse large B-cell non-Hodgkin lymphoma (DLBC-NHL), on account of the strong immunosuppression due to the disease itself, cytotoxic drugs, corticosteroids, and especially anti-CD20 immunotherapy, the risk of OBI reactivation is high and has been reported to occur in 3% to 25% of patients, depending on the pharmacological and geographical settings (13–17). In these cases, patients have titratable HBsAg and HBV-DNA in the serum, and as soon as the immune surveillance is reconstituted at the end of chemotherapy, acute hepatitis could arise from simple lobular hepatitis with alanine ammino transferase (ALT) elevation and only minimal lesions to fulminant liver failure and death (18–20).

For this setting of patients with high-risk DLBCL, there is still no consensus among recent guidelines on whether a pre-emptive approach or primary antiviral prophylaxis is the best solution in reducing the risk of OBI reactivation and, thus, immunochemotherapy (ICHT) disruption. Nonetheless, regarding primary antiviral prophylaxis, the length and type of drug is a matter still unresolved (20–23).

We report a prospective series of HBsAg−/HBcAb+ patients receiving long-term lamivudine (LAM) prophylaxis against HBV reactivation concurrently with six courses of R-CHOP-21 (24) (cyclophosphamide, doxorubicin, vincristine, prednisone, and rituximab) remission induction for advanced stage DLBCL. We then compared the LAM efficacy and safety rates in these patients with those of a historical cohort treated with short-term LAM prophylaxis and another historical cohort undergoing a pre-emptive approach.

## Material and methods

### Study design and oversight

**Figure 1:**
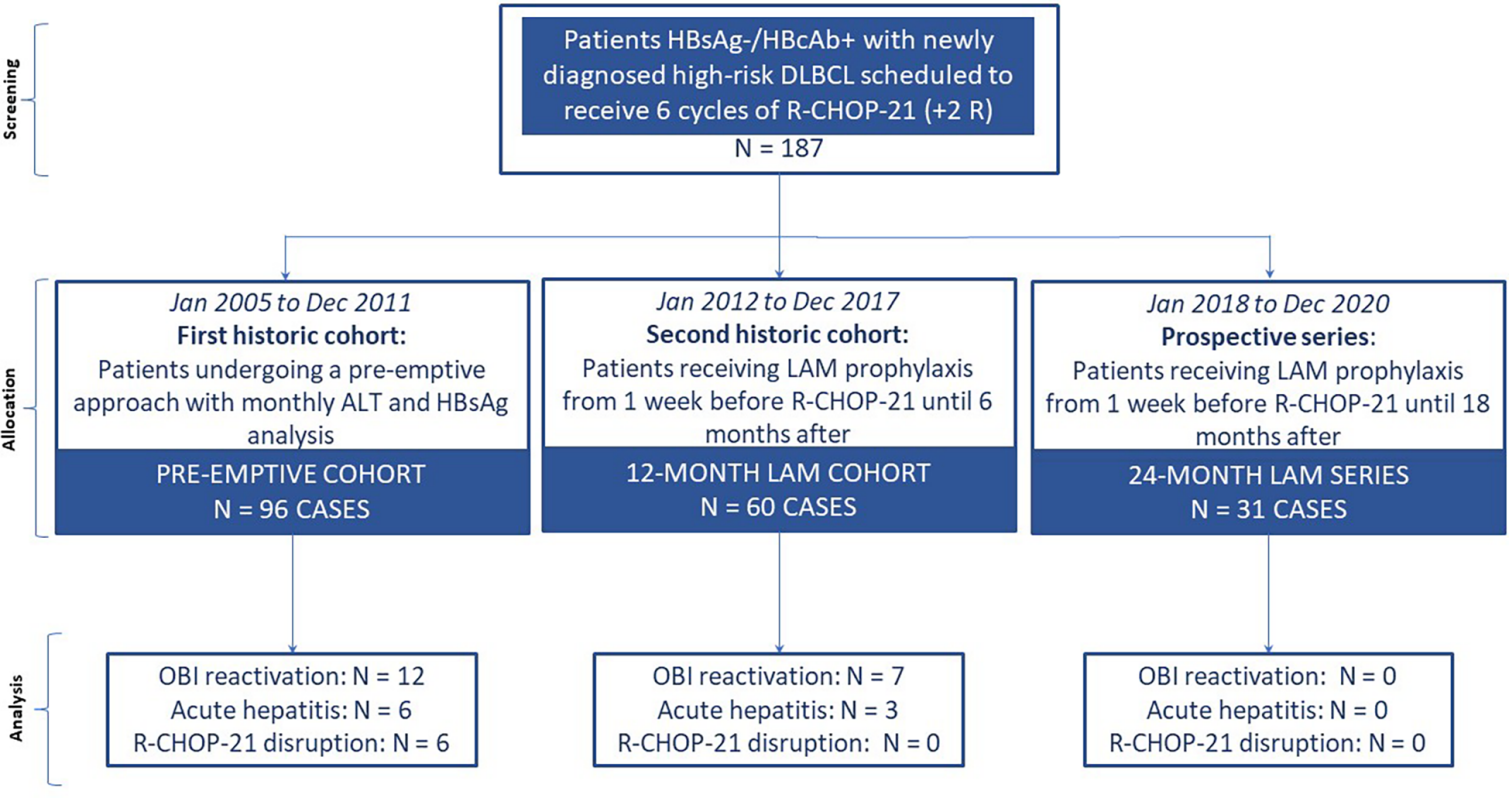
Study flow of the participants.

This was a single-center study conducted in a university hospital in Italy. We carried out a retrospective evaluation from the registry database of the Hematology Unit of the Federico II University of Naples selecting the medical records of HBsAg−/HBcAb+ patients with DLBCL who were scheduled to receive six courses of R-CHOP-21+2R together with i) a pre-emptive antiviral prophylaxis approach from January 2005 to December 2011 (pre-emptive cohort) or ii) a 12-month-long LAM prophylaxis approach from January 2012 to December 2017 (12-month LAM cohort). These two historic cohorts were then compared with a prospective series of patients with the same clinical and serological characteristics consecutively enrolled in the Hematology Unit of the Federico II scheduled to receive six courses of R-CHOP-21+2R and 24-month-long LAM prophylaxis (24-month LAM series) (**Figure 1**).

The Ann Arbor classification stage was determined for all patients at the onset of the neoplastic disease by physical examination, fluorodeoxyglucose (FDG)-positron emission tomography (PET)/total body computed tomography (CT) scan, and bone marrow biopsy, and the International Prognostic Index (IPI) risk score was also calculated (25). The Cheson’s criteria were used to define the response to antineoplastic treatment (26).

Each patient of the prospective series signed the informed consent, while, given the retrospective nature of the two historic cohorts, the acquisition of the informed consents was waived. The study was approved by the Ethics Committee of the Federico II University of Naples (Italy).

### Participants and eligibility criteria

The inclusion criteria were as follows: 1) age ≥18 years, 2) HBsAg−/HBcAb+ serological profile, 3) histological diagnosis of DLBCL reporting morphological and immunohistochemical features (as assessed retrospectively, for the two historic cohorts only, by at least three expert hematopathologists with >10 years of experience in hematopathological analysis) according to the WHO 2016 classification (27), 4) advanced Ann Arbor stage, 5) scheduled six courses of frontline R-CHOP-21 (24) plus two additional rituximab [i.v. cyclophosphamide [750 mg/m^2^], doxorubicin [50 mg/m^2^], vincristine (1.4 mg/m^2^, maximum dose 2 mg), and rituximab [375 mg/m^2^] on day 1 and oral prednisolone (100 mg) on days 1-5, administered every 21 days], and 6) at least 18 months of follow-up from the end of ICHT.

The exclusion criteria were the presence of any hematological malignancy other than DLBCL, previous immunosuppressive treatments of any kind (organ transplant, autoimmune therapies, chemotherapy for other malignancies), and hepatitis D virus (HDV), HCV, and HIV co-infection.

#### First historic cohort enrollment

In the pre-emptive cohort, all patients were scheduled to receive up-front R-CHOP-21+2R for six courses from January 2005 to December 2011 and underwent a pre-emptive approach for OBI reactivation according to the internal hospital guidelines of that historic period. The median follow-up was 68 months (range, 3-120 months). All patients of this cohort were monitored for OBI reactivation with monthly ALT and HBsAg analysis and throughout 18 months after the end of the ICHT scheme. If the patient experienced an ALT derangement more than twice the upper normal value (UNV) and/or an HBsAg seroconversion, complete blood tests were performed to search for liver-bound viruses: HBV-DNA, Epstein–Barr virus-DNA, HCV-RNA, cytomegalovirus-DNA, and herpes virus-RNA 1 and 2 assays were investigated. In case of reactivation, treatment with an antiviral agent was promptly employed.

#### Second historic cohort enrollment

In the 12-month LAM cohort, patients from January 2012 to December 2017 underwent OBI reactivation prophylaxis with LAM 100 mg/day per os from 1 week prior to the ICHT start until 6 months after. HBV monitoring consisted in monthly ALT and HBsAg analysis and 3-monthly HBV-DNA analysis for 12 months after the completion of prophylaxis.

#### Prospective series enrollment

From January 2018 to December 2020, we prospectively enrolled consecutive HBsAg−/HBcAb+ patients admitted to the Hematology Division of Federico II University of Naples for the treatment of newly diagnosed DLBCL with adverse prognostic factors. Patients were scheduled to undergo OBI reactivation prophylaxis with LAM 100 mg/day per os 1 week before the ICHT start until 18 months after the completion (24-month LAM cohort). HBV monitoring consisted in monthly ALT and HBsAg analysis and 3-monthly HBV-DNA analysis during the primary antiviral prophylaxis.

### Virological profile assay

For all patients enrolled, complete serological data of routine biochemistry assays and of HBsAg, HBcAb, HBV envelope antigen (HBeAg), HBe antibody (HBeAb), HBsAb, hepatitis C virus (HCV)-Ab, and HBV-DNA with real-time PCR were available at baseline and follow-up.

HBsAg, HBcAb, and HBsAb were determined by conventional commercial assay kits (Abbott Germany; HBsAg EIA, Abbott, North Chicago). All HBcAb-positive samples were assayed for serum HBV-DNA by a commercial qualitative target amplification method (Cobas AmpliScreen Systems, Roche Molecular, Branchburg, NJ, USA). In order to achieve the highest sensitivity allowed by this method (20 IU/ml), testing was performed on each individual sample without pooling and increasing the volume for extraction (500 μl). The specimens that resulted positive were further tested by a quantitative method (Cobas Amplicore HBV monitor, Roche Molecular System, Branchburg, NJ, USA) to determine the viral load.

### Efficacy analysis

Efficacy analysis of the antiviral strategy focused primarily on ICHT disruption (defined as premature termination of ICHT or a delay >7 days between chemotherapy cycles) related to HBV reactivation (defined as HBsAg seroconversion and/or increase of serum HBV-DNA by at least 1 log above the lower limit of detection of the assay in a person who had previously undetectable HBsAg and HBV-DNA in serum or more than 1 log increase in people who had detectable HBV-DNA at baseline) and hepatitis flare (>3-fold increase in serum ALT that exceeded the reference range) and secondarily on OBI reactivation and HBV-related acute hepatitis flare incidence during follow-up (28–30).

### Safety analysis

The clinical databases of all patients from the historic cohorts were screened for any side effects recorded related to ICHT treatment and/or prophylaxis with the antiviral agent LAM (the latter only for the 12-month LAM cohort). In the prospective series, all patients were investigated regarding possible side effects related to ICHT and/or LAM treatment. In particular, the renal function, hepatic function, and lactic acid levels were monitored every 3 months from the LAM prophylaxis start until completing a period of 24 months in total, due to the notified LAM-related side effects (31) and to the possibility of the emergence of drug-resistant mutants, which could be sequentially followed by virologic breakthrough, hepatitis flare, and even hepatic decompensation. All side effects were reported and graded according to the common terminology criteria for adverse events (CTCAE v.5.0) (32).

### Statistical analysis

All statistical analyses were performed using SPSS software (version 18.0 for Windows; SPSS, Chicago, IL, USA). Student’s *t*-test and the Mann–Whitney *U* test were performed to compare continuous variables, and the *χ^2^* with Yates correction or Fisher exact test was performed to compare categorical variables. Statistical significance was defined as “*P* ≤ 0.05” in a “two-tailed” test with 95% confidence interval (CI). Numerical data were expressed as median and range. The relative risk (RR) was calculated between groups of prophylaxis. Kaplan–Meier for the event-free survival (EFS) for the efficacy analysis was calculated, and the log-rank test for comparison among cohorts was performed. The EFS was calculated from the ICHT start to 24 weeks after (the last administration of rituximab) or until the event of ICHT disruption related to OBI reactivation.

## Results

### Participants

One hundred eighty-seven patients fulfilling the inclusion criteria were enrolled in this case-cohort study. All patients had a diagnosis of DLBCL at high risk with at least one adverse prognostic factor and were scheduled to receive frontline six courses of R-CHOP-21+2R. Each patient received a strong antimicrobial prophylaxis according to the hospital management guidelines including granulocyte colony-stimulating factor (G-CSF), sulfamethoxazole/trimethoprim, and acyclovir (33). The characteristics of the study population are depicted in **Table 1** according to the cohort of prophylaxis. In total, 53% were men and the median age was 59 years (range, 21-84 years). Most of the patients enrolled had ECOG PS 0-2 (94%) and 41% had stage III. No significant differences were found among the groups regarding clinical and disease characteristics.

**Table 1:** Patients’ characteristics at baseline according to the cohorts of prophylaxis against OBI reactivation.

Variable	Total,n (%)	Pre-emptive cohort, n (%)	12-month LAM cohort, n (%)	24-month LAM series, n (%)	P-value
Time period		Jan 2005-Dec 2011	Jan 2012-Dec 2017	Jan 2018-Dec 2020	
Num. of patients	187 (100%)	96 (100%)	60 (100%)	31 (100%)	
Male sex	100 (53%)	52 (54%)	30 (50%)	18 (58%)	0.7
Age, median (range%)	59 (21-84%)	63 (53-74%)	60 (21-74%)	65 (43-84%)	0.9
Histological diagnosis: DLBCL	187 (100%)	96 (100%)	60 (100%)	31 (100%)	
Histological subtypes					0.9
GC	43 (23%)	21 (22%)	14 (23%)	8 (26%)	
N-GC	25 (13%)	11 (11%)	9 (15%)	5 (12%)	
NOS	119 (64%)	64 (67%)	37 (62%)	18 (58%)	
ECOG (PS) 0-2	176 (94%)	92 (96%)	57 (95%)	27 (87%)	1.4
ECOG (PS) 3	11 (6%)	4 (4%)	3 (5%)	4 (13%)	0.9
Ann Arbor stage					0.37
III	77 (41%)	37 (39%)	29 (48%)	11 (35%)	
IV	110 (59%)	59 (61%)	31 (52%)	20 (65%)	
B Symptoms	94 (50.5%)	49 (51%)	29 (48%)	16 (52%)	0.9
Bulky disease	93 (49.5%)	51 (53%)	28 (47%)	14 (45%)	0.6
IPI> 3	41 (22%)	21 (22%)	15 (25%)	5 (16%)	0.1
Front line ICHT: R-CHOP-21+2R	187 (100%)	96 (100%)	60 (100%)	31 (100%)	

Values are n (%) unless otherwise specified.

R-CHOP-21+2R: Rituximab, Cyclophosphamide, Hydroxydaunorubicin, Vincristine and Prednisone (6 cycles of 21 days) and 2 additional Rituximab administration every 21 days (24).

DLBCL, diffuse large B cell lymphoma.

ECOG PS, Eastern Cooperative Group Performance Status.

Ann Arbor staging: stage I defined as one lymph node group; stage II defined as two lymph node groups on one side of the diaphragm; stage III, defined as multiple lymph node groups on both sides of the diaphragm; stage IV, defined as multiple extra-nodal sites or lymph nodes and extra-nodal disease (26).

B symptoms: fever, weight loss >10% in the last 6 months, nocturnal sweat.

Bulky disease: lymph node mass with long axis >5 cm.

IPI: international prognostic index including age greater than 60 years, Ann Arbor stage III or IV, elevated serum LDH, more than 1 extra-nodal site involved (25).

### Pre-emptive cohort patients’ characteristics

In the pre-emptive cohort (from January 2005 to December 2011, the first historic cohort), 96 HBsAg−/HBcAb+ naive DLBCL patients scheduled to receive R-CHOP-21+2R were enrolled. The median age was 63 years (range, 53-74), 53% had bulky disease, and 61% had stage IV. All patients complied with the pre-emptive prophylaxis strategy with a median follow-up of 68 months (range, 3-120 months). OBI reactivation [median serum HBV-DNA level 3.5 × 10^6^ IU/ml (range 1.6–9.1 × 10^6^ IU/ml)] occurred in 12 out of 96 patients (12%) with a median time from the start of ICHT of 24 weeks (range, 12-72 weeks). The clinical and serological characteristics of the patients experiencing OBI reactivation are reported in **Table 2**. Hepatitis flare-up occurred in 6 out of 12 patients, and the median time from diagnosis of OBI reactivation to the occurrence of hepatitis was 8 days (range, 0-18 days). The median value of the highest ALT levels during hepatitis flares was 338 mU/ml (range, 90-800 mU/ml) with severe hepatitis [ALT levels higher than 10-fold of the upper limit of normal (ULN)] occurring in five patients. In particular, six patients experienced ICHT disruption after a median time of 24 weeks (12-24 weeks) from treatment start: two patients, for severe hepatitis, terminated prematurely the R-CHOP-21+2 R scheme (after 5 and 3 cycles, respectively), while the other four patients delayed the ICHT scheme for a median time of 14 days (range, 7-21 days) and two of them also reduced the dose of the chemotherapy agents (**Table 2**) but completed the R-CHOP-21+2 R scheme. All patients with OBI reactivation started treatment with lamivudine 100 mg daily per os and continued it for a median time of 35 weeks experiencing HBV-DNA recovery in a median time of 5 weeks (except one patient who did not recover but died because of DLBCL progression). At the end of treatment evaluation (26), three out of the six patients experiencing ICHT disruption recorded a complete remission, one patient recorded a stable disease (14 days of delay and ICHT dose intensity reduced), one patient that terminated prematurely after 5 cycles achieved a partial remission, while the other one did not complete the treatment for severe hepatitis after only 3 cycles and died due to disease progression. Thus, in this cohort, the complete hematological remission rate was 77%.

**Table 2:** Clinical and serological characteristics of patients experiencing OBI reactivation in the pre-emptive cohort and in the 12-month LAM prophylaxis cohort.

Patients no.	Age (y)	Sex	Stage	Cohort ^§^	Baseline			Time of diagnosis of OBI reactivation	At OBI reactivation		Type of antiviral therapy	Antiviral therapy duration	Time to HBV recovery (weeks)	Follow up HBsAg status
					HBsAg (+/-)	HBV-DNA (IU/ml)	ALT (U/L)	weeks after ICHT start	HBV-DNA (x10^6^ UI/ml)	Peak ALT (X N.V)	LAM/TDF	weeks	weeks after antiviral therapy	+/-
1	64	F	III	Pre-emptive	–	Neg	23	40	3,5	2.2x	LAM	28	8	–
2 *	73	M	II	Pre-emptive	–	Neg	18	24	4.5	10x	LAM	36	4	–
3 *	64	F	IV	Pre-emptive	–	Neg	25	12	6.5	12x	LAM	22	3	–
4	71	M	IV	Pre-emptive	–	Neg	31	24	1,6	11x	LAM	48	3	–
5	62	M	IV	Pre-emptive	–	Neg	34	52	1,3	2.1x	LAM	34	8	+
6 *	66	M	II	Pre-emptive	–	Neg	29	20	1.7	2.7x	LAM	41	5	+
7	59	M	III	Pre-emptive	–	Neg	34	72	1,6	2.4x	LAM	38	3	+
8	54	F	IV	Pre-emptive	–	Neg	32	32	3.1	2.8x	LAM	34	3.8	+
9 *	71	F	III	Pre-emptive	–	Neg	22	14	5.5	14x	LAM	Until death	3.5	–
10	58	F	IV	Pre-emptive	–	Neg	24	60	3,4	2x	LAM	25	5	+
11 *	53	M	IIII	Pre-emptive	–	Neg	26	18	2.2	3.2x	LAM	48	4.2	–
12 *	53	F	III	Pre-emptive	–	Neg	19	48	9.1	20x	LAM	44	6	–
13	63	F	III	12-month LAM	–	Neg	32	52	2,1	7x	TDF	24	4.5	–
14	80	F	III	12-month LAM	–	Neg	25	60	8,4	5x	TDF	20	6	–
15	72	M	IV	12-month LAM	–	Neg	30	58	4,8	3x	TDF	24	7	+
16	67	F	IV	12-month LAM	–	Neg	16	72	5,2	2.7x	TDF	22	6.5	+
17	56	M	III	12-month LAM	–	Neg	21	48	3,9	2.8x	TDF	42	3,8	+
18	48	F	II	12-month LAM	–	Neg	29	40	1,9	4.3x	TDF	38	5	–
19	40	F	II	12-month LAM	–	Neg	21	60	4,5	2x	TDF	30	5.5	–

LAM, Lamivudine; TDF, Tenofovir disoproxil fumarate; HBsAg, Hepatitis B surface antigen; HBV-DNA, Hepatitis B Virus-DNA; ALT, alanine transaminase.

§ No reactivation events were registered in the 24-month LAM series.

* Patients with OBI reactivation experiencing R-CHOP-21 (24) disruption. For patient n° 2 and 9, ICHT was prematurely terminated; for patient n° 3, both Hydroxydaunorubicin (H) and Vincristine (O) dose was reduced by 25% (with 14 days of delay), whereas for patient n° 11, H and O dose was reduced by 25% and 20%, respectively (with 16 days of delay); for patients n° 6 and 12, ICHT was delayed by 7 and 21 days, respectively, and resumed without dose modification.

OBI reactivation: occult hepatitis B virus infection reactivation defined as HBsAg seroconversion and HBV-DNA detectable in the serum (>2000 IU/mL); Acute hepatitis: ≥3-fold increase in serum AST that exceeded the reference range; ICHT disruption: premature termination of immune-chemotherapy (ICHT) of R-CHOP21+2R or delay ≥7 days between immune-chemotherapy cycles or any modification of dose density/intensity (28).

In the 12-month LAM cohort and 24-month LAM series, HBV monitoring consisted in monthly ALT and HBsAg analysis and 3-monthly HBV-DNA analysis for 12 months after prophylaxis end. In the pre-emptive cohort, HBV monitoring consisted in monthly ALT and HBsAg analysis.

### Twelve-month LAM cohort patients’ characteristics

In the 12-month LAM cohort (from January 2012 to December 2017), 60 HBsAg−/HBcAb+ DLBCL naive patients were enrolled; the median age was 60 years (range, 21-74 years) and 52% had stage IV. All patients received R-CHOP-21+2R as scheduled and the median follow-up was 45.5 months (range, 13-118 months). Prophylaxis consisted in LAM 100 mg daily per os starting 1 week before the R-CHOP-21+2R scheme until 6 months after the completion of the ICHT scheme. OBI reactivation [median serum HBV-DNA level 5 × 10^6^ IU/ml (range 2.1-8.4 10^6^ IU/ml)] occurred in 7 out of 60 patients (10%) during follow-up after a median time of 58 weeks (range, 40-72 weeks) from the ICHT start; thus, no ICHT disruption events were recorded. The clinical and serological characteristics of the patients experiencing OBI reactivation are reported in **Table 2**. Overall, three out of seven patients experienced hepatitis flare-up with a median time from OBI reactivation to the occurrence of hepatitis of 7 days (range, 0-16 days). No severe hepatitis was recorded with a median value of the highest ALT levels during hepatitis flares of 110 mU/ml (range, 68-220 mU/ml). All patients experiencing OBI reactivation started treatment with tenofovir disoproxil fumarate (TDF) 245 mg daily per os and continued it for a median time of 24 weeks experiencing HBV recovery (HBV-DNA-negative in serum) in a median time of 6 weeks. Thus, in this cohort, the complete hematological remission rate was 80%.

### Twenty-four-month LAM series patients’ characteristics

In the prospective series of the 24-month LAM long prophylaxis, 31 HBsAg−/HbcAb+ patients with newly diagnosed DLBCL were enrolled from January 2018 to December 2020. The overall median age was 65 years (range, 43-84 years), 65% had stage IV, and 45% had bulky disease. All patients received R-CHOP-21+2R as scheduled and the median follow-up was 34 months (range, 24-48 months). Prophylaxis consisted in LAM 100 mg daily per os from 1 week before R-CHOP-21+ 2R until 18 months after anti-lymphoma therapy. No events of OBI reactivation, hepatitis, and ICHT disruption were recorded. Thus, in this cohort, the complete hematological remission rate was 87%.

### Efficacy analysis

**Figure 2:**
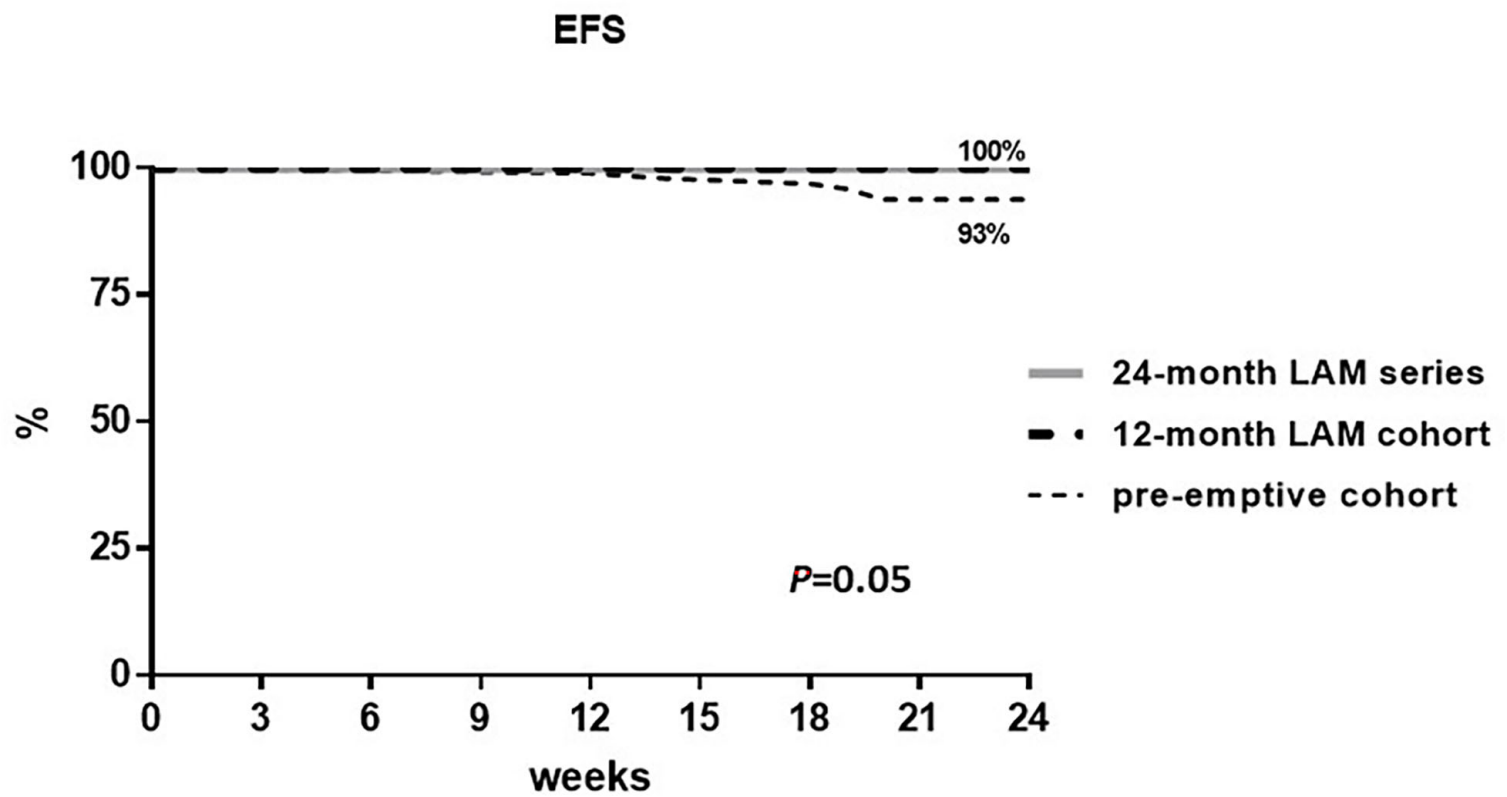
Event-free survival (%) for immunochemotherapy (ICHT) disruption.

Overall, in the total population, only six patients experienced ICHT disruption related to OBI reactivation, while no other causes of ICHT disruption were recorded in all the groups. In particular, all patients experiencing ICHT disruption were from the pre-emptive cohort with an ICHT disruption rate of 6% (6 out of 96 patients) versus 0% in the 12-month LAM cohort and in the 24-month series. The EFS for ICHT disruption at 24 weeks from the R-CHOP-21+2R scheme start was 93% in the pre-emptive cohort and 100% in the 12-month LAM cohort and in the 24-month LAM series. The log-rank test for the group comparison was calculated and resulted as statistically significant with a *P*-value of 0.05 (**Figure 2**).

OBI reactivation incidence was 12% in the pre-emptive cohort, 10% in the 12-month LAM cohort, and 0% in the 24-month LAM series (*P* = 0.12). Hepatitis was recorded in 6% of the patients in the pre-emptive cohort, in 5% of the patients in the 12-month LAM cohort, and in 0% of the patients in the 24-month LAM series (*P* = 0.37). No statistical difference among the three groups was found for OBI reactivation and hepatitis, but the pairwise *χ* ^2^ comparisons were significant for OBI reactivation in the pre-emptive cohort versus the 24-month LAM series [12% *vs*. 0% (*P* = 0.04)] and for the 12-month LAM cohort versus the 24-month LAM series [10% *vs*. 0% (*P* = 0.04)], while there was no significant difference between the pre-emptive cohort and the 12-month LAM cohort [12% *vs*. 10% (*P* = 0.6; RR = 1.2, 95% CI 0.4-3.6)] and for the pairwise *χ* ^2^ comparisons of hepatitis.

### Safety analysis

All patients’ side effects related to LAM were of low/moderate intensity (grade 1 or 2) and did not lead to discontinuation of LAM treatment or to ICHT disruption. In particular the, 18% (11/60) of the patients in the 12-month-LAM cohort and 22% (7/31) of the patients in the 24-month LAM cohort experienced fatigue (*P* = 0.21); headache was observed in the 23% (14/60) and 26% (8/31) of the patients, respectively (*P* = 0.25); nausea was observed in 12% (7/60) and 16% (5/31) of the patients, respectively (*P* = 0.08); diarrhea was recorded in 15% (9/60) and 16% (5/31) of the patients, respectively (*P* = 0.09); and abdominal pain was recorded in 17% (10/60) and 10% (3/31) of the patients (*P* = 0.09), respectively. No renal failure and lactic acidosis events were recorded.

## Discussion

Our single-center survey showed that OBI reactivation in patients with advanced DLBCL undergoing frontline six courses of R-CHOP-21+2R remains a clinically relevant problem, especially if an inadequate prophylaxis strategy is employed. The endpoint of the study was chosen specifically to evaluate the efficacy of a longer period of primary antiviral prophylaxis with LAM 18 months after R-CHOP-21+2R end in preventing not only ICHT disruption but also late events of OBI reactivation and hepatitis in HBsAg−/HBcAb+ patients undergoing standard ICHT for high-risk DLBCL.

In this delicate setting of patients, it is crucial that the treating physician renders the patient in the best performance status in order to continue the scheduled antilymphoma strategy. OBI reactivation and related hepatitis may negatively affect the hematological disease cure, leading to disruption of the dose-density/dose-intensity program against DLBCL (34). On the other hand, late events of HBV-related transaminase flares may negatively affect morbidity.

Worldwide, there is still no consensus regarding the optimal approach (pre-emptive or primary antiviral prophylaxis) for preventing OBI reactivation in HBsAg−/HBcAb+ patients, and in choosing antiviral prophylaxis strategy, the optimal duration of prophylactic antiviral therapy and the type of antiviral drug remain to be defined (19, 20).

Several studies suggest a pre-emptive approach in HBsAg−/HBcAb+ lymphoma patients monitored with close on-demand antiviral therapy in case of OBI reactivation while receiving rituximab (11, 35), and even if in some studies HBV reactivation did not lead to an increase in mortality/morbidity, it still remains a life-threatening risk that can endanger the completion of the ICHT scheme for patients with high-risk hematological disease (36).

The Italian Association for Liver Research (AISF) issued recommendations of a universal prophylaxis in subjects treated with intense immunosuppression (i.e., protocols including monoclonal antibodies and/or strongly immunosuppressive therapies, i.e., “dose-dense” regimens) but without indications of the type of prophylaxis and duration. Notably, these recommendations had low strength (B and C) and were derived from retrospective studies (35). According to the recent American Association for the Study of Liver Diseases (AASLD) and the European Association for the Study of the Liver (EASL) clinical practice guidelines, HBsAg and anti-HBc testing should be performed in all patients before initiation of any immunosuppressive, cytotoxic, or immunomodulatory therapy (21, 22). These guidelines support a pre-emptive approach in some cases but lean toward a prophylaxis approach for patients receiving rituximab. Notably, antiviral prophylaxis is recommended for HBsAg−/HBcAb+ subjects for 12 months when rituximab is employed, and anti-HBV drugs with a high-resistance barrier [entecavir (ETV), TDF, or tenofovir adenosil fumarate] should be preferred (21, 22). However, drugs with a high-resistance barrier are characterized by a concerning safety profile (31). In particular, there have been concerns for renal toxicity with their use (e.g., Fanconi syndrome and diabetes insipidus) and bone density changes (37–39).

Nonetheless, the costs of a longer period of prophylaxis are consistent with newer agents with respect to LAM, and it should be taken into account the numerous drug interactions with TDF and ETV (40, 41). To the best of our knowledge, this is the first study with an adequate sample size of 187 patients providing data regarding a comparison of three different strategies of prophylaxis in three homogeneous cohorts of HBsAg-/HBcAb+ patients with high-risk DLBCL undergoing a standardized ICHT scheme.

We showed in a prospective series that the primary prophylaxis strategy with LAM for 18 months after R-CHOP-21+2R end (24 months in total) is the most effective and significantly reduces the risk of ICHT disruption for OBI reactivation. As a matter of fact, in a pairwise comparison, we showed the superiority of the 24-month-long prophylaxis versus the 12-month-long and pre-emptive strategy in reducing the incidence of OBI reactivation (*P* = 0.04 for both comparisons), while no difference was found in comparing the two historic cohorts.

Calculating the number needed to treat (NNT) (42), seven patients would have to receive the 24-month-long LAM prophylaxis to not have one OBI reactivation event. LAM resulted to have a potent antiviral activity against HBV in our series and was inexpensive, safe, and well-tolerated since the majority of patients reported mild adverse events and no patients suffered from complications of grade >2 according to the CTCAE in both groups receiving LAM.

Nonetheless, the possibility of emergence of drug-resistant mutants, which could be sequentially followed by a virologic breakthrough, has not arisen in the prospective series with a longer LAM treatment. As a matter of fact, in the majority of patients of the 12-month LAM cohort, OBI reactivation occurred shortly after LAM suspension due to the persistence of viral replication in NHL HBV-infected subjects that have not already recovered the immune surveillance function. It appears that a longer period (18 months after rituximab treatment) is needed for re-establishing tolerance to a small viral load (43).

Our study had some limitations. First, this is a single-center trial, and therefore, studies from other institutions are required to evaluate on a larger scale the proposed prophylaxis scheme. Second, our study is a case-cohort study comparing a prospective analysis with two cohorts characterized by a retrospective nature risking a bias of selection. Third, the sample size of the prospective series is relatively small in comparison with the two retrospective cohorts. Probably, this is the reason why no escapes were recorded in the prospective series since the incidence of escape rate was relatively small (3.3%) in the retrospective cohort. Fourth, the follow-up period of the 24-month LAM series is relatively short (median follow-up of 24 months), especially in comparison with the other two cohorts; thus, we could not perform a survival analysis. As a matter of fact, a further concern is the effect of HBV reactivation on the disease outcome of patients. Although some patients experiencing ICHT disruption presented a worse outcome at disease re-evaluation, the patient number in this study and the follow-up of the prospective series were too small to form any conclusions.

In conclusion, the results of this study indicate that in patients with DLBCL and OBI, HBV reactivation induced by R-CHOP-21+2R is not uncommon and clinically significant. Regular monitoring with prompt antiviral therapy upon reactivation is an approach that resulted in a higher incidence of OBI reactivation and related hepatitis causing ICHT disruption. A short primary prophylaxis period with LAM can be adopted, but patients’ serum HBV-DNA had to be very closely monitored to avoid hepatitis flares shortly after the end of prophylaxis. For these reasons, a longer period of 24 months of LAM primary prophylaxis appeared to be very effective and safe in this setting of patients at high risk with hematological disease who received full dosages of chemotherapy agents, steroids, and rituximab.

## Data availability statement

The original contributions presented in the study are included in the article/supplementary material. Further inquiries can be directed to the corresponding author.

## Ethics statement

The studies involving human participants were reviewed and approved by the Ethical Committee of Università Federico II-Cardarelli. The patients/participants provided their written informed consent to participate in this study.

## Author contributions

CG designed the research, performed the research, and wrote the paper. MP designed the research and wrote the paper. A.V, DA, LD, MG, CF, MC, AS, GC, EV, MM, and GT collected the data. CG and NP analyzed the data. FP and MP performed the final revision of the manuscript. All authors contributed to the article and approved the submitted version.
